# Kinome-Wide RNAi Screen Uncovers Role of Ballchen in Maintenance of Gene Activation by Trithorax Group in *Drosophila*

**DOI:** 10.3389/fcell.2021.637873

**Published:** 2021-03-05

**Authors:** Muhammad Haider Farooq Khan, Jawad Akhtar, Zain Umer, Najma Shaheen, Ammad Shaukat, Muhammad Shahbaz Munir, Aziz Mithani, Saima Anwar, Muhammad Tariq

**Affiliations:** Department of Biology, Syed Babar Ali School of Science and Engineering, Lahore University of Management Sciences, Lahore, Pakistan

**Keywords:** trithorax group, polycomb group, kinases, NHK-1, transcriptional memory, histone modifications, Ballchen

## Abstract

Polycomb group (PcG) and trithorax group (trxG) proteins are evolutionary conserved factors that contribute to cell fate determination and maintenance of cellular identities during development of multicellular organisms. The PcG maintains heritable patterns of gene silencing while trxG acts as anti-silencing factors by conserving activation of cell type specific genes. Genetic and molecular analysis has revealed extensive details about how different PcG and trxG complexes antagonize each other to maintain cell fates, however, the cellular signaling components that contribute to the preservation of gene expression by PcG/trxG remain elusive. Here, we report an *ex vivo* kinome-wide RNAi screen in *Drosophila* aimed at identifying cell signaling genes that facilitate trxG in counteracting PcG mediated repression. From the list of trxG candidates, Ballchen (BALL), a histone kinase known to phosphorylate histone H2A at threonine 119 (H2AT119p), was characterized as a trxG regulator. The *ball* mutant exhibits strong genetic interactions with *Polycomb* (*Pc*) and *trithorax* (*trx*) mutants and loss of BALL affects expression of trxG target genes. BALL co-localizes with Trithorax on chromatin and depletion of BALL results in increased H2AK118 ubiquitination, a histone mark central to PcG mediated gene silencing. Moreover, BALL was found to substantially associate with known TRX binding sites across the genome. Genome wide distribution of BALL also overlaps with H3K4me3 and H3K27ac at actively transcribed genes. We propose that BALL mediated signaling positively contributes to the maintenance of gene activation by trxG in counteracting the repressive effect of PcG.

## Introduction

In metazoans, specialization of cell types that make up an organism is linked to cell type specific gene expression patterns established during early development. In order to maintain specialized state, the particular expression profile of genes needs to be transmitted to daughter cells through successive mitotic divisions in all cell lineages, a phenomenon termed as transcriptional cellular memory. Maintenance of transcriptional cellular memory and consequent cellular identity involves a combinatorial act of various epigenetic mechanisms, such as DNA methylation, histone modifications, non-coding RNAs, and chromatin remodeling (Cavalli and Heard, 2019). Genetic analysis in *Drosophila* uncovered two groups of evolutionarily conserved genes, namely Polycomb group (PcG) and trithorax group (trxG), responsible for maintaining stable and heritable states of gene repression and activation, respectively (Jürgens, 1985; Breen and Duncan, 1986; Kennison and Tamkun, 1988). Molecular analysis revealed that proteins encoded by the PcG and trxG act in large multi-protein complexes, and modify the local properties of chromatin to maintain expression patterns of their target genes. Both groups exert their functions by binding to chromosomal elements known as PREs (polycomb response elements) and by interacting with histones and transcription machinery (Kassis et al., 2017; Cavalli and Heard, 2019). The PcG complexes, PRC1 and PRC2 (polycomb repressive complex 1 and 2), are known to maintain repression by ubiquitination of histone H2A at lysine 118 (H2AK118ub1) (Wang et al., 2004) and methylation of histone H3 at lysine 27 (H3K27me) (Francis et al., 2001; Cao et al., 2002; Czermin et al., 2002), respectively. In contrast to PcG, trxG is more heterogeneous and comprises of proteins that activate transcription by modifying histone tails or remodeling chromatin (Schuettengruber et al., 2017). Despite their diversity, one cellular function that unifies trxG proteins is their role in counteracting PcG mediated gene silencing.

The fact that trxG and PcG coexist at the chromatin regardless of the expression states of their target genes suggests that PcG and trxG not only compete with each other to regulate transcriptional states but also associate with their target genes as dynamic complexes (Breiling et al., 2001; Dellino et al., 2004; Klymenko and Müller, 2004; Papp and Müller, 2006; Beisel et al., 2007). Although, the chromatin structure and modifications appear to play a fundamental role in maintenance of transcriptional cellular memory, the signal that favors PcG or trxG to either repress or activate gene expression state remains elusive. It is plausible to assume that cell signaling pathways are of prime importance due to their ability to respond to intra and extracellular changes as well as their capacity to influence nuclear factors involved in gene repression or activation. Cell signaling components, especially the protein kinases, control a repertoire of cellular processes by modifying more than two-third of cellular proteins (Ardito et al., 2017). Interestingly, both the PcG and trxG complexes lack kinases. In *Drosophila*, FSH is the only kinase present in canonical trxG members. However, being an atypical kinase, with no known kinase domain (Chang et al., 2007), FSH performs its cellular functions *via* its bromodomain and interaction with ASH1 (Kockmann et al., 2013). Although different cellular processes linked to epigenetic inheritance, such as maintenance of chromosomal architecture and transcription (Stadhouders et al., 2019), are regulated by protein kinases (Nowak and Corces, 2000, 2004), the role of cell signaling components in maintaining gene activation by trxG or repression by PcG remains elusive.

Here, we report an RNA interference (RNAi) based reverse genetics screen to identify cell signaling proteins that contribute to the maintenance of gene activation by trxG. An *ex vivo* kinome-wide RNAi screen was carried out using a well-characterized reporter in *Drosophila* cells (Umer et al., 2019). The primary RNAi screen led to the identification of 27 cell signaling genes that impaired expression of the reporter similar to *trx* and *ash1* upon knockdown. The majority of candidates in the list were protein kinases, but regulatory subunits of kinase complexes, kinase inhibitors, nucleotide kinases and lipid kinases were also present. Importantly, the presence of FSH, the only trxG member with predicted kinase activity, in the list of candidates validated the functionality of our screen. From the list of candidates obtained in the primary screen, nine serine-threonine kinases were further confirmed in a secondary screen which affected reporter system similar to the effect of TRX and ASH1 depletion.

Next, we performed genetic and molecular analysis of Ballchen (BALL), a histone kinase in the list of candidate genes, and showed that BALL is required to maintain gene activation by trxG. BALL mutant exhibits trxG like behavior by strongly suppressing extra sex comb phenotype caused by *Pc* mutations as well as by enhancing the homeotic phenotype in *trx* mutants. This strong genetic interaction between *ball* and trxG is substantiated by a drastic reduction in expression of homeotic and non-homeotic targets of trxG in *ball* mutant embryos. Importantly, reduced expression of trxG targets due to depletion of BALL also correlates with enhanced levels of H2AK118ub1, a histone modification central to PcG mediated gene repression (Blackledge et al., 2014, 2020; Kalb et al., 2014; Tamburri et al., 2020). Finally, the genome-wide binding sites of BALL identified in *Drosophila* S2 cells by ChIP-seq revealed BALL occupancy at transcription start sites (TSS) and at the majority of known TRX binding sites. Since BALL is known to phosphorylate H2A threonine 119 (H2AT119p) (Aihara et al., 2004), our data supports the notion that BALL contributes to maintenance of gene activation by counteracting PRC1 mediated H2AK118ub1.

## Results

### Kinome-Wide RNAi Screen Discovered Kinases Affecting Cell Memory Maintenance

**FIGURE 1:**
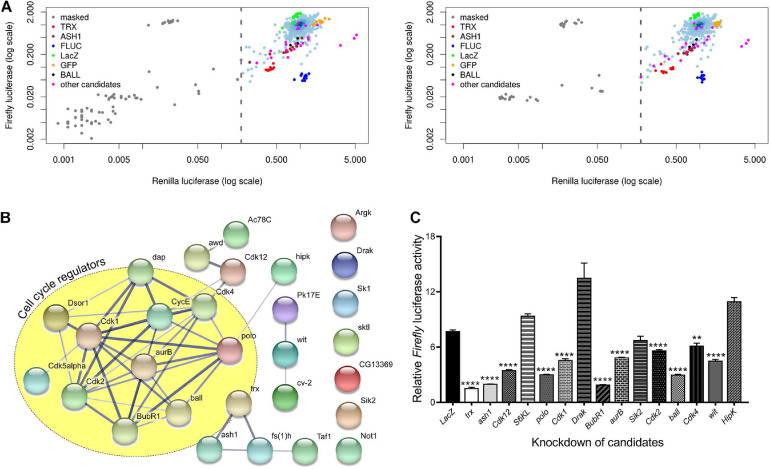
Kinome-wide RNAi screen data analysis and validation. **(A)** Scatterplots of the plate median corrected intensity values for *Firefly* luciferase (F.Luc) against the plate median corrected intensity values for *Renilla* luciferase (R.Luc). To generate a list of trxG candidate genes that affect gene activation, Z-scores of *trx* (red) and *ash1* (maroon) knockdowns were used to set cut-off value which is represented by a dashed line. Knockdown of *trx* (red) and *ash1* (maroon) as well as knockdown of *F.Luc* (blue) were used as positive controls. Cells treated with dsRNA against *LacZ* (green) and *GFP* (orange) served as negative controls. Genes affecting activity of both F.Luc and R.Luc were masked (gray) and were not investigated further. *Ball* is shown in black whereas the remaining eight candidates which were reproduced in the secondary screen are shown in magenta. Data shown represents two independent experiments of the kinome-wide RNAi screen. **(B)** Protein-protein interaction network of candidate genes generated using STRING database (Snel, 2000; Szklarczyk et al., 2019). Nodes depict proteins that are connected by lines of varying thickness. Thickness of lines demonstrates the degree of confidence for the interaction between connected nodes. The yellow circle is marking candidates that are known to associate with cell cycle regulation. A confidence level of 0.400 was used to generate the interaction map. **(C)** D.Mel-2 cells treated with dsRNA against selected serine-threonine kinases from the list of candidate genes from primary screen were transfected with the experimental reporter (*F.Luc*) and invariant co-reporter (*R.Luc*). Knockdown of all but four kinases resulted in significantly lower relative *Firefly* luciferase activity when compared to cells treated with dsRNA against *LacZ*. Cells treated with dsRNA against *trx* and *ash1* were used as positive control. Experiment was performed in triplicate and independent *t*-tests were done for statistical analysis (**p ≤ 0.01, or ****p ≤ 0.0001).

To identify the role of cell signaling genes in the maintenance of gene activation by trxG, we used a previously characterized cell-based reporter *PBX-bxd-IDE-F.Luc* (hereafter referred to as *PRE-F.Luc*). In this reporter, the *Firefly* luciferase gene is under the control of *Drosophila Ubx* (*Ultrabithorax*) promoter and *bxd PRE* along with *PBX* (*postbithorax*) and *IDE* (*Imaginal Disk Enhancer*) enhancers of *Ubx*. The sensitivity and specificity of this reporter to the changing levels of PcG and trxG are already described (Umer et al., 2019). Using this reporter, we performed an *ex vivo* RNAi screen, covering all known and predicted kinases and their associated proteins from the HD2 dsRNA library (Horn et al., 2010). Each gene was knocked down in triplicates and the entire experiment was performed twice. dsRNAs against known trxG members (*trx, ash1*) and the reporter gene (*F.Luc*) were used as positive controls, whereas dsRNA against *LacZ* and *GFP* were used as negative controls in all plates. *Drosophila* cells treated with dsRNAs were co-transfected with *PRE-F.Luc* reporter and actin promoter-driven *Renilla* luciferase (*R.Luc*) which was used as a normalization control (Figure 1A and Supplementary Figure 1). Since *R.Luc* is driven by a constitutive promoter, it also served as a control to exclude the genes that may affect general transcriptional machinery. Five days after transfection, the activity of both *F.Luc* and *R.Luc* was determined and Z-scores were calculated. A list of potential trxG regulators was generated (Table 1) based on the Z-scores obtained from positive controls (*trx, ash1*) which were used as threshold to define the cut-off for candidates. Of 400 genes that were screened, 27 candidates specifically resulted in reduced *PRE-F.Luc* expression, an effect similar to the knockdown of *trx* and *ash1*. The only trxG member with predicted kinase activity, *fs(1)h* was also found among the top hits of the screen (Table 1). Moreover, proteins having known genetic and molecular interactions with trxG also appeared in the list of candidates. For instance, human CDK1 is known to phosphorylate EZH2, the enzymatic subunit of PRC2, at threonine 487 and as a consequence decreases its methyltransferase activity in mesenchymal stem cells (Wei et al., 2011). In addition to protein kinases, lipid kinases, nucleotide kinases, kinase inhibitors and regulatory subunits of kinase complexes were also present in the list of candidates. Gene ontological analysis using STRING (Figure 1B) and PANTHER (Supplementary Table 1) databases revealed that most candidates are localized in the nucleus and are involved in cell cycle regulation. For example, CDK2 and its associated Cyclin E are present in the cell cycle regulators cluster that also biochemically interact with trxG proteins, Brahma (BRM) and Moira (MOR), the core proteins of Brahma associated protein complex (Brumby et al., 2002).

**TABLE 1:** List of candidate genes along with their respective Z-scores, annotation symbols, human orthologs, and a summary of their known functions.

**Candidate genes**	***Z*-score**	**Annotation symbol**	**Summary of known functions/general description.**	**Human orthologs**	**Validation in secondary screen**
Cyclin-dependent kinase 12 (Cdk12)	16.45	CG7597	Hyperphosphorylates the C- terminal heptapeptide domain of RNA Pol-II.	CDK12, CDK13	Yes
Sphingosine kinase 1 (Sk1)	15.27	CG1747	Converts sphingosine to sphingosine 1-phosphate.	SPHK1, SPHK2	Not checked
CG13369	13.17	CG13369	Ribokinase	RBKS	Not checked
Female sterile (1) homeotic [fs(1)h]	10.72	CG2252	Involved in pattern formation by regulating homeotic genes expression.	BRD2, BRD3, BRD4, BRDT	Not checked
S6 Kinase Like (S6KL, Pk17E)	10.62	CG7001	Promotes proteasomal degradation of BMP receptor tkv thus inhibits BMP signaling.	RSKR	No
Polo	9.75	CG12306	Control different aspects of cell division.	PLK1, PLK2, PLK3	Yes
Abnormal wing disks (awd)	9.7	CG2210	A nucleotide diphosphate kinase, that is involved in the biosynthesis of nucleotide triphosphates.	NME1, NME1-NME2, NME2, NME3, NME4	Not checked
Dacapo	8.49	CG1772	CDK inhibitor from the CIP/KIP family that inhibits the CycE-CDK2 complex.	CDKN1C	Not checked
Crossveinless 2 (cv-2)	8.48	CG15671	A secreted protein that can bind BMPs and their receptor tkv to either inhibit or promote BMP signaling.	BMPER	Not checked
Cyclin-dependent kinase 1 (Cdk1)	8.08	CG5363	Regulates cell cycle progression by phosphorylating hundreds of target proteins.	CDK1	Yes
Death-associated protein kinase-related (Drak)	8.06	CG32666	Involved in the development of epithelial tissues.	STK17B, STK17A	No
Bub1-related kinase (BubR1)	7.93	CG7838	Important for spindle assembly checkpoint during the cell cycle.	BUB1, BUB1B	Yes
Aurora B (aurB)	7.39	CG6620	Known to phosphorylate Histone H3 at serine 10. Cell cycle-related roles include chromosome condensation, kinetochore assembly, and cytokinesis.	AURKB, AURKC	Yes
Adenylyl cyclase 78C (Ac78C)	7.37	CG10564	Catalyzes the synthesis of 3′,5′-cyclic AMP from adenosine triphosphate in response to G-protein coupled receptor signaling.	ADCY8	Not checked
Not1	7.32	CG34407	Poly(A)-specific ribonuclease that is involved in mRNA degradation.	CNOT1	Not checked
Salt-inducible kinase 2 (Sik2)	7.17	CG4290	Important for lipid storage and energy homeostasis.	SIK2, SIK1B, SIK1	No
Cyclin-dependent kinase 2 (Cdk2)	7.13	CG10498	Catalytic subunit of CycE-Cdk2 complex that acts during progression from G1 and S phase of mitosis.	CDK2, CDK3	Yes
Cyclin E (CycE)	6.96	CG3938	A member of the cyclin group of proteins that act as a regulatory subunit of CycE-Cdk2 complex.	CCNE1, CCNE2	Not checked
Cdk5 activator-like protein (Cdk5alpha)	6.63	CG5387	Regulatory subunit of Cdk5-Cdk5α complex that is active in neurons.	CDK5R1, CDK5R2	Not checked
TBP-associated factor 1 (Taf1)	6.55	CG17603	Binds with initiator elements at transcription start sites. Part of TFIID, an evolutionary conserved multimeric protein complex involved in general transcription.	TAF1, TAF1L	Not checked
Downstream of raf1 (Dsor1)	6.55	CG15793	Dual specificity kinase that acts as MAPKK. It is activated by Raf.	MAP2K1, MAP2K2	Not checked
Arginine kinase (Argk)	6.51	CG32031	Belongs to the ATP: guanidino phosphotransferase family.	CKM, CKB, CKMT1A, CKMT2	Not checked
Ballchen (ball)	6.44	CG6386	A nucleosomal histone kinase that phosphorylates Histone H2A at threonine 119.	VRK1, VRK2, VRK3	Yes
Cyclin-dependent kinase 4 (Cdk4)	6.32	CG5072	Essential for cell cycle progression and promotes cellular growth.	CDK6, CDK4	Yes
Wishful thinking (wit)	6.28	CG10776	BMP type II receptor that regulates neurotransmission at the neuromuscular junction and synaptic homeostasis.	BMPR2, AMHR2	Yes
Homeodomain interacting protein kinase (hipk)	6.27	CG17090	A member of the DYRK family of kinases that contributes to several different signaling pathways including Wingless, Notch, Hippo, JNK, and cell death.	HIPK1, HIPK2, HIPK3, HIPK4	No
Skittles (sktl)	6.15	CG9985	Converts phosphatidylinositol 4-Phosphate (PIP) into phosphoinositol-4,5-bisphosphate (PIP4,5).	PIP5K1A	Not checked

*The *Z*-score of six was used as a threshold based on *trx* and *ash1* scores and genes above this cut-off were selected as candidate trxG genes. Results for validation of candidates in secondary screen are mentioned (Yes/No) in the last column. Candidates which were not included for further analysis in the secondary screen are listed as “Not checked.” General description of each gene is based on information obtained from UniProt and FlyBase databases.*

Since serine/threonine phosphorylations make up the bulk of signaling milieu in a cell, we selected serine/threonine protein kinases from the list of candidates and performed a secondary screen using *PRE-F.Luc* reporter (Figure 1C). To avoid any library specific bias, we generated dsRNAs for our secondary screen using a library different from the ones used in the primary screen (see section “Materials and Methods”). Cells treated with dsRNA against *trx* and *ash1* were used as positive controls while cells treated with dsRNA against *LacZ* served as a negative control. Depletion of nine out of the thirteen selected kinases resulted in significantly decreased relative activity of *F.Luc*. Finally, we chose *ballchen* (*ball*) from the list of candidate genes to explore its genetic and molecular link with trxG because it is a known histone kinase which phosphorylates histone H2AT119 (Aihara et al., 2004). Depletion of BALL drastically reduced expression of the reporter gene, relative F.Luc activity, when compared with other candidates in the secondary screen (Figure 1C). Moreover, it is also known to be involved in both cell cycle (Fiona Cullen et al., 2005) and signal transduction pathways (Herzig et al., 2014; Yakulov et al., 2014), making it the most suitable representative gene of the candidate list.

### *Ball* Exhibits trxG Like Behavior

**FIGURE 2:**
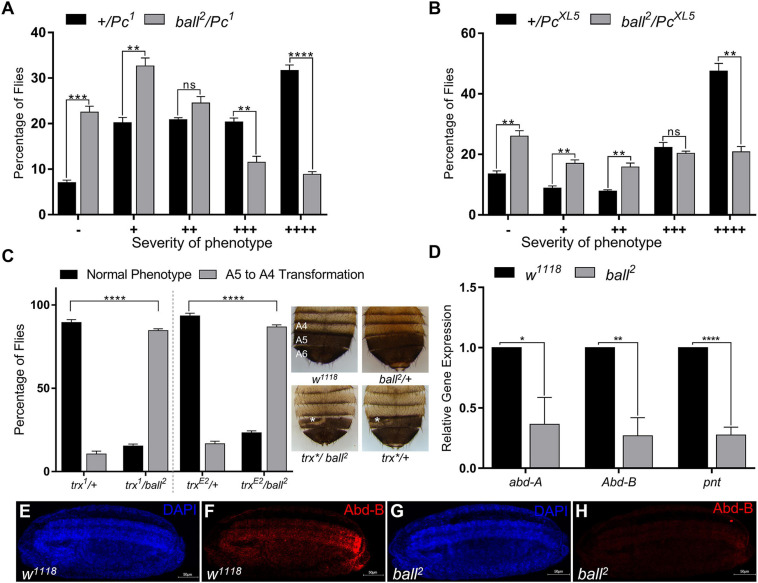
*ballchen* mutation exhibits trxG like behavior. **(A,B)** Ballchen mutant flies (*ball*^2^) were crossed to two different alleles of *Pc* (*Pc*^1^ and *Pc*^*XL5*^). *Pc* alleles (*Pc*^1^ and *Pc*^*XL5*^) crossed to *w*^1118^ flies were used as control crosses. Heterozygous *Pc/*+males, *Pc*^1^/+ **(A)** and *Pc*^*XL5*^/+ **(B)**, from control crosses exhibit strong extra sex comb phenotype. In contrast, *ball*^2^ strongly suppressed the extra sex comb phenotype in both *ball^2^/Pc^1^* **(A)** and *ball^2^/Pc^*XL5*^* **(B)** male flies. 200 male flies were analyzed for each cross and data shown represents two independent experiments. Male flies were categorized according to the severity of extra sex comb phenotype. These categories are: –, no extra sex combs; +, 1–2 hairs on 2nd leg; ++, more than three hairs on 2nd leg; +++, more than 3 hairs on 2nd leg and 1–2 hairs on 3rd leg; ++++, strong sex combs on both 2nd and 3rd pairs of legs as described previously (Tariq et al., 2009). **(C)** *ball*^2^ mutant flies were crossed to two different alleles of *trx* (*trx*^1^, *trx*^*E2*^). A cross between *trx* mutants and *w*^1118^ served as a control. Males from the resulting progeny were scored for A5 to A4 transformation (loss of pigmentation in A5, marked by asterisk), a known *trx* mutant phenotype. *trx*/+heterozygotes from the cross of *w*^1118^ with *trx* mutants were used as control. Compared to the control, *ball*^2^/*trx*^1^ and *ball*^2^/*trx*^*E2*^ showed a higher percentage of flies with A5 to A4 transformation, indicating a strong enhancement of *trx* mutant phenotype. Representative images of *w*^1118^, *ball*, and *trx* heterozygous mutants and *ball/trx* double mutants are shown. The expressivity of A5 to A4 transformation phenotype of trx*/ball^2^ was comparable to trx*/+. All crosses were carried out in triplicates and independent *t*-tests were performed for analyzing each category (*p ≤ 0.05, **p ≤ 0.01, ***p ≤ 0.001, or ****p ≤ 0.0001). **(D)** Significantly low levels of *abd-A*, *Abd-B* and *pnt* expression was detected through qRT-PCR in homozygous *ball*^2^ embryos when compared with *w*^1118^ embryos. Independent *t*-tests were performed for each gene analysis (*p ≤ 0.05, **p ≤ 0.01, ***p ≤ 0.001, or ****p ≤ 0.0001). **(E–H)** Immunostaining of stage 15 embryos with Abd-B antibody is shown in *w*^1118^ **(E,F)** as well as homozygous *ball*^2^ **(G,H)** embryos. As compared to *w*^1118^ **(F)**, *ball*^2^ embryos showed strongly diminished Abd-B **(H)** expression.

To investigate whether *ball* genetically interacts with the PcG/trxG system, *ball* mutant flies were crossed to two different mutant alleles of *Pc* (*Pc*^1^, *Pc*^*XL5*^). *Pc* heterozygous mutants display a strong extra sex comb phenotype in males (Lewis, 1978). The *ball* mutant (*ball*^2^) strongly suppressed this extra sex comb phenotype (Figures 2A,B) which supports the role of *ball* as a trxG-like factor controlling homeotic phenotype. Next, we examined the genetic interaction of *ball* with *trithorax* (*trx*) by crossing *ball*^2^ with two different alleles of *trx* (*trx*^1^ or *trx*^*E2*^) mutants. As compared to wild-type flies, *trx* heterozygous males show loss of pigmentation on the 5^th^ abdominal segment, referred to as A5 to A4 transformation (Ingham and Whittle, 1980; Figure 2C). However, *ball^2^/trx trans-*heterozygotes exhibit a significantly higher percentage of flies with A5 to A4 transformations (Figure 2C) as compared to *trx* heterozygotes indicating increased penetrance of *trx* phenotype. The suppression of extra sex comb phenotype and the enhancement of *trx* phenotype by *ball* mutant indicates that *ball* exhibits a trxG like behavior.

Next, we investigated the effect of *ball* mutation on the expression of homeotic and non-homeotic targets of PcG/trxG. Since homozygous *ball*^2^ mutants do not develop into adults (Herzig et al., 2014), we used homozygous *ball*^2^ embryos to analyze mRNA levels of *abd-A, Abd-B*, and *pnt* through real-time PCR (Figure 2D). As compared to *w*^1118^ embryos, a significant reduction in expression of *abd-A, Abd-B*, and *pnt* was observed. Since *Abd-B* is the gene responsible for pigmentation in A5 and A6 abdominal segments in males (Jeong et al., 2006), its depletion in *ball*^2^ mutant embryos correlates with the loss of pigmentation in *ball^2^/trx trans-*heterozygotes. Importantly, immunostaining of stage 15 homozygous *ball*^2^ embryos with Abd-B antibody further validated the effect of *ball* mutation on the expression of *Abd-B* (Figures 2E–H). At this stage of development in wild-type embryos, Abd-B expression progressively increases from PS10-14 (Figure 2F). However, in *ball*^2^ mutants, Abd-B expression is drastically reduced (Figure 2H) which correlates with significantly diminished *Abd-B* mRNA levels in homozygous *ball*^2^ embryos (Figure 2D). Together with genetic evidence, these results suggest that trxG requires BALL for maintenance of gene activation during development.

### BALL Co-localizes With TRX and Inhibits PRC1

**FIGURE 3:**
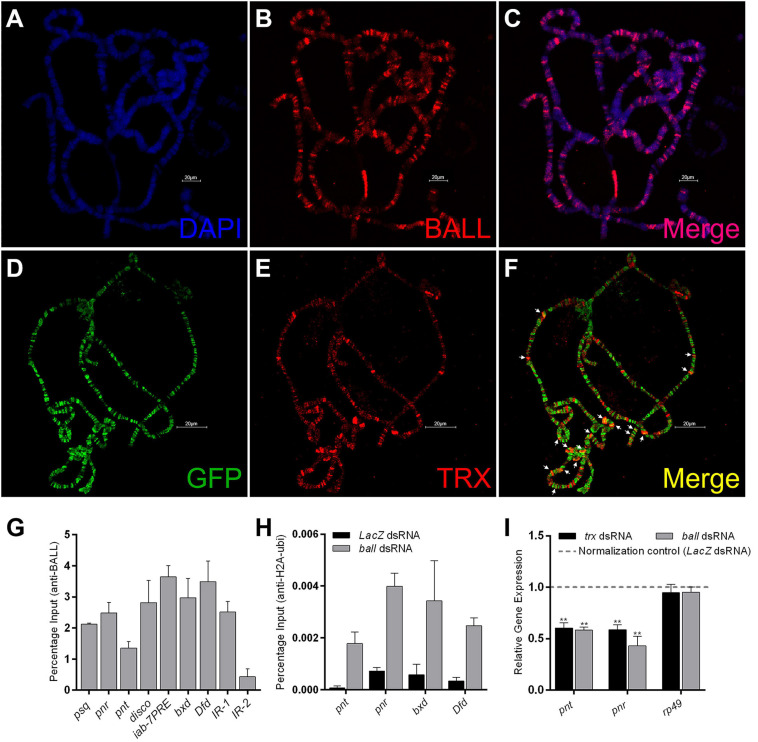
BALL co-localizes with TRX at the chromatin and antagonizes PcG. **(A–C)** Polytene chromosomes from third instar larvae stained with DAPI **(A)** and BALL antibody **(B)**. **(D–F)** Third instar larvae expressing GFP-tagged BALL were stained with GFP **(D)** and TRX **(E)** antibodies. Co-localization of BALL and TRX is clearly seen at several loci [**(F)**, white arrows]. **(G)** Strong enrichment of BALL at non-homeotic (*psq, pnr, pnt*, and *disco*) and homeotic (*iab-7PRE*, *bxd*, *Dfd*) trxG targets was observed by ChIP from wild-type *Drosophila* S2 cells using BALL antibody. *IR-1* and *IR-2* are intergenic regions not bound by TRX and served as controls (Papp and Müller, 2006). **(H)** ChIP from BALL depleted cells showed enhanced H2AK118ub1 levels at non-homeotic (*pnt*, *pnr*) and homeotic (*bxd*, *Dfd*) targets of trxG as compared to cells treated with dsRNA against *LacZ*. **(I)** Using qRT-PCR, relative gene expression of *pnt* and *pnr* was analyzed in cells after *ball* or *trx* knockdown. *LacZ* dsRNA treated cells were used as normalization control (dashed line) and the expression of *pnt* and *pnr* in *ball* and *trx* depleted cells was plotted. BALL depleted cells showed a significantly decreased expression of *pnt* and *pnr* similar to the cells with *trx* knockdown. The mRNA levels of a ribosomal protein, *rp49*, served as a negative control. Independent *t*-tests were performed for analyzing relative expression of each gene (*p ≤ 0.05, **p ≤ 0.01, ***p ≤ 0.001, or ****p ≤ 0.0001).

Although BALL is known to bind chromatin during mitosis, its association with chromatin during interphase is not yet established. Here we show by immunostaining of polytene chromosomes that BALL associates at numerous chromosomal sites (Figures 3A–C). Next, to investigate if BALL and TRX co-localize on chromatin, *UAS-ball-EGFP* transgenic flies were crossed to a salivary gland specific GAL4 driver line (*sgs-gal4*). Immunostaining of polytene chromosomes from salivary glands of third instar larvae expressing *ball-EGFP* revealed a partial overlap between BALL and TRX (Figures 3D–F). Chromatin association of BALL at TRX binding sites was validated by ChIP analysis from S2 cells using BALL antibody (Figure 3G). BALL was found at the TSS of *pipsqueak* (*psq*), *pannier* (*pnr*), *pointed* (*pnt*), and *disconnected* (*disco*), known binding sites of PcG/trxG. Moreover, BALL was also observed at *iab-7PRE*, *bxd*, and *Dfd* regulatory regions of homeotic genes. Besides the association of BALL at homeotic and non-homeotic genes, we also analyzed two intergenic regions, *IR-1* and *IR-2*, which are not bound by TRX (Papp and Müller, 2006). BALL was found to occupy *IR-1* but not *IR-2*, which served as a negative control for our ChIP data.

Since BALL is known to phosphorylate histone H2AT119, a residue adjacent to H2AK118, we questioned whether the H2AT119ph mark is inhibitory for H2AK118ub1, an established hallmark of PcG mediated gene repression (Blackledge et al., 2014, 2020; Kalb et al., 2014; Tamburri et al., 2020). To this end, we performed ChIP from cells where *ball* was knocked down using RNAi (Supplementary Figure 2). BALL depleted cells exhibited an increased H2AK118ub1 at both transcriptionally active (*pnt*, *pnr*) and repressed (*bxd*, *Dfd*) targets of trxG as compared to cells treated with dsRNA against *LacZ* which served as a control (Figure 3H). To investigate if the increase in H2AK118ub1 is also reflected in the expression of trxG target genes, we analyzed the mRNA levels of transcriptionally active genes (*pnt* and *pnr*) through qRT-PCR in BALL depleted cells. The depletion of BALL indeed resulted in decreased expression of *pnt* and *pnr* as compared to cells treated with dsRNA against *LacZ* (Figure 3I). These results suggest that BALL is required by trxG to maintain gene activation and it may counteract PcG by inhibiting H2AK118ub1, a histone modification catalyzed by PRC1 subunit dRING, and in turn shift the balance in favor of trxG.

### Genome-Wide Binding Profile of BALL Correlates With TRX and Gene Activation

**FIGURE 4:**
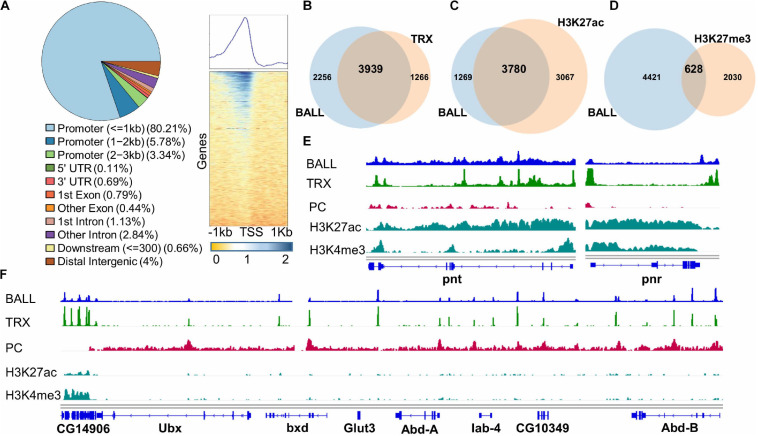
Genome-wide binding profile of BALL analyzed by ChIP-seq from *Drosophila* S2 cells. **(A)** ChIPseeker was utilized to generate a pie chart that represents distribution of BALL across different regions of the genome. BALL is enriched predominantly in promoter (=1 kb) regions followed by promoter (1–2 kb) regions, distal intergenic regions and promoter (2–3 kb) regions. Heat map [**(A)**, right panel] shows enrichment of BALL within 1 kb upstream of TSS of genes across the genome correlating with 80% occupancy of BALL in the promoter regions as shown in pie chart. **(B–D)** Lists of associated annotated peaks were generated for comparative analysis shown by Venn diagram analyses which show co-occupancy of BALL on known TRX peaks **(B)** and association of BALL with H3K27ac **(C)** and H3K27me3 **(D)** marked genes. **(E)** Genome Browser view of BALL enrichment compared to that of TRX, PC, H3K27ac, and H3K4me3 at *pnt* and *pnr* genes. **(F)** Genome Browser view of ChIP-seq data showing binding profile of BALL and its comparison with TRX, PC, H3K27ac, and H3K4me3 across the bithorax complex (BX-C). Highly enriched BALL and TRX correlates with high levels of both H3K27ac and H3K4me3 and absence of PC at *CG14906*, a gene present just downstream of *Ubx*. In contrast, PC is highly enriched throughout BX-C locus justifying the silenced state of *Ubx*, *abd-A*, and *Abd-B* genes in *Drosophila* S2 cells. Both BALL and TRX are present at few overlapping binding sites in BX-C.

The association of BALL with chromatin and its co-localization with TRX on polytene chromosomes led us to determine the genome-wide binding sites of BALL. For this purpose, ChIP with BALL antibody was performed using formaldehyde fixed chromatin from *Drosophila* S2 cells and the purified DNA was sequenced. BALL was found to bind a total of 6,195 sites (Figure 4A and Supplementary Table 2), more than 80% of which were in the promoter regions. Further analysis of the genome binding profile revealed that BALL was mostly present up to 1 kb upstream of TSS of genes (Figure 4A). Comparison of BALL enriched peaks to previously published TRX binding profile (Rickels et al., 2016) showed a considerable overlap between the two (Figure 4B). BALL was found to occupy 3,939 of all known TRX binding sites thus signifying the involvement of BALL in transcriptional cellular memory. Besides an overwhelming overlap between BALL and TRX binding sites, a large number (3780) of genes bound by BALL were also found to be marked by H3K27ac (Rickels et al., 2016), a hallmark of gene activation by trxG (Figure 4C; Tie et al., 2009, 2014). In contrast, less than a quarter of genes (628) bound by BALL are marked by H3K27me3 (Rickels et al., 2016), a covalent modification that correlates with repression by PcG (Figure 4D). Since expression of *pnt* and *pnr* genes is reduced after depletion of BALL or TRX, ChIP-seq data was specifically analyzed for the association of BALL and TRX across the genomic regions of *pnt* and *pnr*. High levels of both BALL and TRX at the *pnt* and *pnr* genomic regions coincide with high levels of H3K27ac and H3K4me3 (Rickels et al., 2016) and little to no PC (Kang et al., 2015; Figure 4E). Additionally, analysis of BALL binding sites across the bithorax complex (BX-C) genomic region revealed that BALL binding profile mimics TRX binding in BX-C (Figure 4F). Since genes within BX-C are silent in S2 cells, their repression correlates with the high prevalence of PC enrichment throughout BX-C and only a few overlapping BALL and TRX binding sites. Importantly, an actively transcribed gene, CG14906, just downstream of *Ubx* in BX-C shows highly enriched BALL and TRX association that correlates with both H3K4me3 and H3K27ac. All these results indicate a close association between BALL and TRX which suggests an important role for BALL in maintenance of gene activation governed by trxG.

## Discussion

Our results demonstrate the success of our reverse genetics approach in identifying cell signaling genes that impact trxG mediated gene activation. With the help of a robust cell based reporter, we initially identified 27 candidate genes from the primary kinome-wide RNAi screen in *Drosophila*. This list of candidate genes includes cell signaling kinases and associated genes which were finalized using a stringent criterion based on the Z-scores of *trx* and *ash1* knockdowns used as threshold. Presence of *fs(1)h*, the only trxG member with predicted kinase activity, among the candidate genes validated the robustness of our screen in identifying kinases regulating trxG dependent gene activation. FSH protein facilitates transcription by recognizing acetylated histone marks through its bromodomain and interaction with ASH1 (Kockmann et al., 2013). Other interacting partners of trxG, including *skittles* (*sktl*), *Cyclin E* (*CycE*), and *Cdk2*, were also present in the list of candidate genes. SKTL, a nuclear phosphatidylinositol 4-phosphate 5-kinase, is known to interact with trxG member ASH2 (Cheng and Shearn, 2004) and its catalytic product, phosphoinositol-4,5-bisphosphate (PIP4,5), also stabilizes mammalian Brahma associated factors (BAF) complex on the chromatin (Zhao et al., 1998). CycE and its associated kinase CDK2 are also known to interact with BRM and MOR, two known members of trxG, which form core of the chromatin remodeling BAP complex (Brumby et al., 2002).

Protein kinases are known to affect epigenetic cellular memory by causing phosphorylation of a repertoire of epigenetic regulators including PcG and trxG proteins. For instance, CDK1, a candidate gene in our list, phosphorylates several proteins, including MLL2, LSD1, G9a, SUV39H2, SETD2, DOT1L, p300, KDM2A, and HDAC6, involved in histone modifications and transcription regulation. Besides, DNA maintenance methyltransferase (DNMT1) and the proteins that reverse DNA methylation, i.e., Tet1 and Tet2, are also phosphorylated by CDK1 (Michowski et al., 2020). Additionally, the identification of cell cycle regulators in our screen (Figure 1B and Supplementary Table 1) supports the notion that PcG/trxG system is regulated in a cell cycle dependent manner (Laprell et al., 2017; Asenjo et al., 2020), which may play a role in faithful inheritance of epigenetic states across cell divisions.

*Drosophila* BALL was found to be one of the strongest candidates among the nine serine/threonine kinases validated in the secondary screen. Genetic and molecular characterization of BALL described here has revealed a novel role for BALL in transcriptional cellular memory. Although, BALL is a known histone kinase that modifies histone H2AT119 (Aihara et al., 2004), it had not been associated with the maintenance of gene activation by trxG prior to our kinome-wide RNAi screen. Our results revealed a strong genetic interaction of *ball* mutant with *Pc* and *trx* mutations. We demonstrated that *ball* mutant exhibits trxG like behavior as it strongly suppresses extra sex comb phenotype of *Pc* mutants. Moreover, mutation in *ball* enhances *trx* mutant phenotype, which correlates with the strong suppression of extra sex comb phenotype. This trxG-like behavior of *ball* is substantiated by reduced expression of trxG target genes in *ball* homozygous mutant embryos.

When compared with an overwhelming overlap between BALL and TRX genome-wide binding profiles in *Drosophila* Schneider cells, BALL and TRX colocalization was observed on a fewer sites on polytene chromosomes. This can be explained by the fact that polytene chromosomes are very specialized type of chromosomes that originate specifically in salivary glands. Nevertheless, presence of BALL together with TRX at majority of sites in ChIP-seq analysis suggests that trxG requires BALL for maintenance of gene activation. Since BALL phosphorylates histone H2AT119, a residue adjacent to H2AK118, which is ubiquitinated by dRING in PRC1, it is plausible to assume that BALL mediated phosphorylation may counteract H2AK118ub1 by PRC1 and facilitate trxG. Additionally, enhanced levels of H2AK118ub1 found at trxG targets when the function of BALL was compromised in cells is in agreement with the downregulation of trxG target genes. All the results presented here support the notion that BALL mediated phosphorylation of H2AT119 counteracts H2AK118ub1 (Aihara et al., 2004, 2016) and plays a role in gene activation. Interplay between phosphorylation of specific amino acids and covalent modifications of neighboring lysine residues in histones is reported to play a role in epigenetic gene regulation. For example, H3S28 phosphorylation is known to counteract H3K27me3 and promote H3K27ac in maintenance of gene activation by trxG (Gehani et al., 2010). Similarly, H3S10 phosphorylation is proposed to promote H3K14ac and counteract H3K9me3 and contribute to gene activation (Lo et al., 2000; Fischle et al., 2003). Since the genome-wide binding profile of BALL also correlates with presence of H3K27ac and H3K4me3 on active genes, it is important to investigate if BALL mediated phosphorylation impacts H3K27ac or H3K4me3. Notably, phosphorylated histone H3 threonine 3 (H3T3p), a mitosis specific mark catalyzed by Haspin kinase (Dai et al., 2005) and mammalian VRK1 (homolog of BALL) (Kang et al., 2007), is reported to inhibit demethylation of H3K4me3 (Su et al., 2016). However, elucidation of the mechanistic basis of BALL mediated phosphorylation in maintenance of gene activation by trxG requires further scrutiny at the molecular and biochemical levels. Since BALL plays a role in chromosome condensation during mitosis, it will be interesting to probe if phosphorylation mediated signaling events involving BALL may help in survival of chromatin structures and modifications involved in the maintenance of transcriptional cellular memory during processes of DNA replication and cell division. Based on the discovery of a predominantly large number of cell cycle associated genes in our screen, it will also be interesting to scrutinize if phosphorylation could be the central epigenetic mark that acts as a stable anchor for epigenetic inheritance and helps restore dynamic interactions between PcG and trxG proteins and their target genes after cell division.

## Materials and Methods

### RNAi Screen

HD2 kinome-wide sub-library was used for primary RNAi screen (Horn et al., 2010), details of which can be obtained from^1^. D.Mel-2 cells were incubated with dsRNAs against all known and predicted kinases and associated proteins. In 384 well plates, dsRNAs against each gene was present in triplicates and the entire experiment was performed twice in primary screen. Six 384 well plates were used for each experiment and a final concentration of 50 ng/μl dsRNA was used in each well. The *trx, ash1*, and *F.Luc* specific dsRNAs were used as positive controls whereas dsRNAs against *LacZ* and *GFP* were used as negative control in each plate. 8,000 cells in 30 μl were dispensed per well with a multidrop dispenser. The cells were spun down for 10 s at 900 rpm, the plates were sealed and incubated at 25°C. Next day, *PRE-F.Luc* and *Actin-R.Luc* were transfected in these cells where *R.Luc* served as a normalization control. After 5 days of transfections, relative F.Luc expression was calculated by taking the ratios of the experimental reporter (F.Luc) to the invariant co-reporter (R.Luc). Knockdown of genes that affected both F.Luc and R.Luc, indicating an impact on general transcription, translation or cell survival, were removed from further analysis. Relative F.Luc expressions were averaged for both replicates. Z-scores of *trx* and *ash1* knockdown, 6.13 and 6.15 respectively, were used to define a cut-off of six for shortlisting candidates. The cut-off based on the Z-scores of known trxG members provided a more stringent criterion as compared to using 99.99% confidence values as cut-off. To rule out library specific biases in the secondary screen, primers from the DRSC library instead of the HD2 library were used. Details of DRSC primers can be found at^2^. The secondary screen was performed in 96 well plates with each well having 2 μg dsRNA. Fifty thousand cells in 100 μl were dispensed in each well. Incubation and data acquisition was performed as described above for the primary screen.

### Protein Interaction Analysis

An interaction map of candidates from the primary kinome-wide RNAi screen was generated by using STRING database (Szklarczyk et al., 2019) with a confidence of 0.400. Unclustered nodes were manually aligned. Thickness of the edges indicate the strength of interaction. Gene ontological analysis for protein class enrichment was performed on the candidate list using PANTHER classification system (Mi et al., 2019). All known and predicted kinases and their associated proteins were used as the reference database.

### Fly Strains and Genetic Analysis

The following fly strains were obtained from Bloomington *Drosophila* Stock Center: *Pc^*XL5*^/TM3Ser,Sb, Pc^1^/TM3*Ser, *trx*^1^ (BN 2114), *trx*^*E2*^ (BN 24160). *ball*^2^ was a gift from A. Herzig (Herzig et al., 2014). To obtain homozygous *ball*^2^ embryos, GFP negative embryos were selected from the progeny of *ball*^2^ mutants balanced over constitutively expressing GFP balancer, *P(neoFRT)82B e ball^2^/TM3, P[w(*+*mC)* = *ActGFP]JMR2, Ser(1)* (please also see section “Microscopy”). For immunostaining of polytene chromosomes, *P[w*^+^*^*mC*^UASp-ball.T:Avic/EGFP* = *pballE]2.1* (gift from A. Herzig) was crossed with *P*[*Sgs3-GAL4.PD]* and third instar larvae from the progeny were used. For extra sex comb analysis, mutant *ball*^2^ and *w*^1118^ were crossed to *Pc* alleles (*Pc*^1^ and *Pc*^*XL5*^) at 25°C. Males in the progeny of these crosses were scored for extra sex comb phenotype as described previously (Tariq et al., 2009). *ball*^2^ flies were crossed to *trx* alleles (*trx*^1^ and *trx*^*E2*^) and with *w*^1118^ at 29°C. The progeny of these crosses was scored for *trx* mutant phenotypes. For analyzing the expression of homeotic genes *in vivo*, homozygous *ball*^2^ and *w*^1118^ embryos were stained with antibodies as described previously (Umer et al., 2019).

### Drosophila Cell Culture

Schneider’s *Drosophila* medium (Gibco, ThermoFisher Scientific), supplemented with 10% fetal bovine serum (Gibco, ThermoFisher Scientific) and 1% penicillin–streptomycin (Gibco, ThermoFisher Scientific) was used to culture *Drosophila* S2 cells. Express Five SFM (Gibco, ThermoFisher Scientific) supplemented with 20 mM GlutaMAX (Gibco, ThermoFisher Scientific) and 1% penicillin–streptomycin was used to grow D.Mel-2 cells.

### RNA Isolation and Analysis of Relative Gene Expression

The procedures employed for RNA isolation using TRIzol and cDNA synthesis followed the manufacturer’s instructions (Life Technologies). Analysis of relative gene expression using qPCR was performed as described previously (Umer et al., 2019). Expression of specific genes in test and control samples were normalized against expression of Actin used as an internal control. Relative gene expression was calculated by ΔΔC_T_ method (Schmittgen and Livak, 2008). The test sample was normalized by setting the control sample to one.

### ChIP-qPCR and ChIP-seq Analysis

ChIP using BALL antibody was performed with 3 × 10^7^ *Drosophila* S2 cells. Primer sequences used for qPCR analysis have been described previously (Umer et al., 2019). Sequences of *IR-1* and *IR-2* primers are as follows: *IR-1 Forward* (*CCGAACATGAGACATGGAAAA*), *IR-1 Reverse* (*AAAGTGCCGACAATGCAGTTA*), *IR-2 Forward* (*CAGTTGATGGGATGAATTTGG*), and *IR-2 Reverse* (*TGCCTGTGGTTCTATCCAAAC*). 1 × 10^7^ D.Mel-2 cells were incubated with 10 μg/ml dsRNA for 4 days followed by anti-H2A-ubi ChIP as described previously (Umer et al., 2019). For ChIP-Seq, 3 × 10^7^ *Drosophila* S2 cells were used for ChIP as described above. High throughput sequencing of DNA libraries from ChIP DNA was performed using BGISEQ-500 at BGI Genomics Co., Ltd. Low quality reads and the adaptor sequences were trimmed using BGI SOAPnuke filter (Drmanac et al., 2010; Huang et al., 2017). The sequencing data was uploaded to the Galaxy web public server at^3^ for analysis (Jalili et al., 2020). The filtered data was mapped to *Drosophila* genome (dm6) using Bowtie version 2 at default parameters (Langmead et al., 2009). Peak calling was performed using MACS version 2 with 200 bp read extension and 0.05 minimum FDR (q-value) cut-off for peak detection (Zhang et al., 2008). List of annotated peaks was extracted from peak file using ChIPseeker (Yu et al., 2015). Genome-wide binding profiles for BALL ChIP-seq replicates were generated using the “bamCompare” tool with a bin size of 50. Heat map of the BALL binding profile was generated using the “computeMatrix” and “plotHeatmap” deepTools (Ramírez et al., 2016). Sequencing data have been submitted to the GEO under the accession number (GSE165685). ChIP-seq data of H3K4me3, H3K27ac, H3K27me3, and TRX were taken from GSE81795 (Rickels et al., 2016) while PC ChIP-seq data was downloaded from GSE66183 (Kang et al., 2015).

### Immunostaining

Larvae expressing GFP tagged BALL in salivary glands were obtained by crossing *P[w*^+^*^*mC*^UASp-ball.T:Avic/EGFP* = *pballE]2.1* (gift from A. Herzig) with *P[Sgs3-GAL4.PD]*. Polytene squashes were prepared and immunostained using standard protocol (Sullivan et al., 2000). Dechorionated stage 15 embryos were used for immunostaining using standard protocol (Sullivan et al., 2000).

### Microscopy

For observing extra sex comb phenotype, Olympus SZ51 stereomicroscope was used. To image the loss of abdominal pigmentation phenotype, male flies of the desired genotype were transferred to 70% ethanol to dehydrate. The dehydrated flies were dissected under Olympus SZ51 stereomicroscope on a dissection slide. The abdominal portion of the fly was isolated and rehydrated in water for 5–10 min. After rehydration, abdomens were mounted on a glass slide in Hoyer’s medium (Sullivan et al., 2000). A coverslip was placed over the specimen and was incubated at 65°C for 50 min. Images were acquired using Nikon C-DSS230 epifluorescent stereomicroscope at 3.5 × magnification. The same epifluorescent stereomicroscope was used for the selection of GFP negative embryos (to get homozygous *ball*^2^ mutant embryos) in experiments where immunostaining and real-time PCR analysis of embryos was performed. Imaging of embryos was done at 20 × magnification on Nikon C2 confocal microscope. NIS Elements image acquisition software was utilized for imaging and analysis. All larval dissections for polytene chromosomes were performed using LABOMED CZM6 stereo zoom microscope and Nikon C2 confocal microscope was used for imaging. All polytene chromosomes were visualized at 60 × magnification.

### Antibodies

Following antibodies were used during this study: mouse anti-Abd-B (DSHB, 1A2E9, IF: 1:40), rabbit anti-TRX (gift from R. Paro, IF: 1:20), rabbit anti-BALL (Gift from A. Herzig, IF 1:20, ChIP: 2 μl) mouse anti-GFP (Roche, 11814460001, IF: 1:50), mouse anti-Tubulin (Abcam, ab44928, WB: 1:2,000), mouse anti-FLAG M2 (Sigma Aldrich, WB: 1:2,000, ChIP: 5 μl), mouse anti-H2A-ubi (Millipore, 05-678, ChIP: 5 μl). HRP conjugated secondary antibodies (Abcam) were used at 1:10,000 dilution for Western blotting while Cy3 and Alexa Fluor 488 conjugated secondary antibodies (Thermo Fisher Scientific) were used at 1:100 dilution for immunofluorescence.

## Data Availability Statement

The datasets presented in this study can be found in online repositories. The names of the repository/repositories and accession number(s) can be found in the article/Supplementary Material.

## Author Contributions

MHFK, JA, ZU, SA, and MT designed the research. MHFK, JA, ZU, NS, AS, and SA performed the experiments. MHFK, JA, ZU, NS, and MT wrote the manuscript. AM analyzed the screen data while MSM helped with ChIP-seq data analysis. All authors approved the final version of the manuscript.

## Conflict of Interest

The authors declare that the research was conducted in the absence of any commercial or financial relationships that could be construed as a potential conflict of interest.

## Footnotes

1 http://rnai.dkfz.de

2 https://fgr.hms.harvard.edu/fly-cell-based-rnai

3 http://usegalaxy.org/

## Supplementary Materials

Supplementary Figure 1 Schematic of *ex vivo* kinome-wide RNAi screen and data analysis. **(A)** 384-well plates containing dsRNAs against different genes were loaded with equal number of cells in each well. 24 h after seeding cells, *PRE-F.Luc* and *Actin-R.Luc* were co-transfected and luciferase values were determined 5 days later. **(B)** Box plots representing plate median normalized data for kinome-wide RNAi screen, replicate 1 (left) and replicate 2 (right).

Supplementary Figure 2 Confirmation of *ball* knockdown. Protein lysates were prepared from cells treated with dsRNA against *ball* for 4 days and were analyzed by Western blotting. A drastic decrease in the amount of BALL protein was observed as compared to cells treated with *LacZ* dsRNA.

Supplementary Table 1 Cell cycle specific gene ontology classes are enriched in the list of candidate genes. Analysis was performed using PANTHER classification system with the HD2 kinome subset as reference. Fourth column contains the number of genes expected in the candidate gene list for each GO term based on the reference list. Enrichment of GO terms was calculated by dividing the number of genes in the candidate list belonging to each GO term by its expected number. A *p*-value of 0.05 was used as cut-off to shortlist the GO terms.

Supplementary Table 2 List of peaks generated using ChIPseeker and presented along with their coordinates.
