# Supplementary figures and images for: Kinome-Wide RNAi Screen Uncovers Role of Ballchen in Maintenance of Gene Activation by Trithorax Group in Drosophila
Source: Front Cell Dev Biol. 2021 Mar 5;9:637873. doi: 10.3389/fcell.2021.637873 (PMC7973098; doi:10.3389/fcell.2021.637873)

# Supplementary Figure 1

A

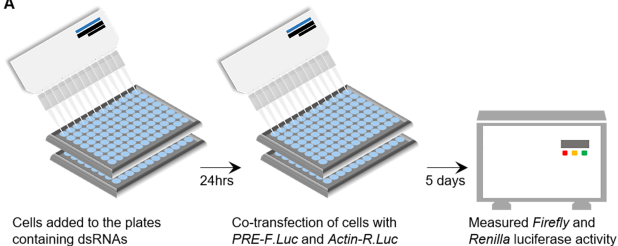

B

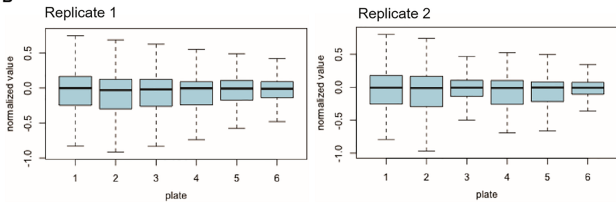

Supplement: Supplementary Figure 1 — Schematic of ex vivo kinome-wide RNAi screen and data analysis. (A) 384-well plates containing dsRNAs against different genes were loaded with equal number of cells in each well. 24 h after seeding cells, PRE-F.Luc and Actin-R.Luc were co-transfected and luciferase values were determined 5 days later. (B) Box plots representing plate median normalized data for kinome-wide RNAi screen, replicate 1 (left) and replicate 2 (right). [file Data_Sheet_1.ZIP › Figure S1.pdf]

# Supplementary Figure 2 dsRNA

*LacZ*

*ball*

Anti-BALL

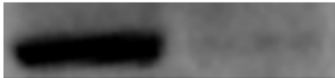

Anti-Tubulin

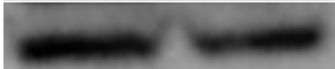

Supplement: Supplementary Figure 1 — Schematic of ex vivo kinome-wide RNAi screen and data analysis. (A) 384-well plates containing dsRNAs against different genes were loaded with equal number of cells in each well. 24 h after seeding cells, PRE-F.Luc and Actin-R.Luc were co-transfected and luciferase values were determined 5 days later. (B) Box plots representing plate median normalized data for kinome-wide RNAi screen, replicate 1 (left) and replicate 2 (right). [file Data_Sheet_1.ZIP › Figure S2.pdf]
